# A giant choledochal cyst in a 17 year old female managed in a resource limited setting: A case report

**DOI:** 10.1016/j.ijscr.2023.108284

**Published:** 2023-05-02

**Authors:** Oscar Atwine, Charles Newton Odongo, Racheal Ainomugisha, Edson Tayebwa, Joshua Muhumuza, Carlos Cabrera Dreque

**Affiliations:** a Department of Surgery, Faculty of Medicine, Mbarara University of Science and Technology, Mbarara, Uganda; b Department of Anatomy, Faculty of Medicine, Soroti University, Soroti, Uganda; c Department of Surgery (Division of Pediatric Surgery), Faculty of Medicine, Mbarara University of Science and Technology, Mbarara, Uganda; d Department of Surgery, Faculty of Clinical Medicine and Dentistry, Kampala international University Western campus, Ishaka-Bushenyi, Uganda; e Department of Surgery, Mubende Regional Referral Hospital, Mubende, Uganda

**Keywords:** Giant choledochal cyst, Resource limited setting, Case report

## Abstract

**Introduction and importance:**

Choledochal cysts are rare congenital bile duct anomalies that lead to cystic dilatations of the biliary tree. This condition is very rare in Africa. When these cysts exceed 10 cm in diameter, they are referred to as giant choledochal cysts, which are much rarer. Giant choledochal cysts present both a diagnostic and surgical challenge. We present a case of a giant Choledochal cyst surgically managed in a resource limited setting with excellent outcome.

**Case presentation:**

A 17-year-old female presented with 4 months history of progressive abdominal distension associated with abdominal pain, yellow discoloration of eyes, and occasional constipation. Abdominal CT-scan revealed a huge cystic mass in the right upper quadrant extending inferiorly to the right lumbar region. Complete excision of a type IA choledochal cyst was done plus cholecystectomy in addition to bilioenteric reconstruction. The patient recovered uneventfully.

**Discussion and conclusion:**

To the best of our knowledge, this is the largest giant Choledochal cyst reported in literature. Even in a resource limited settings, sonography and a CT scan may be all that is required to make a diagnosis. During surgical excision, the surgeon should take extra caution to carefully dissect the adhesions off the giant cyst for a successful complete excision.

## Highlights

• Though giant choledochal cysts are rare, they present a diagnostic and surgical challenge when encountered.
• A thorough abdominal ultrasound scan and CT scan are enough to make a diagnosis.
• Careful dissection of adhesions off the cysts is important for successful complete excision.

## 1 Introduction

Choledochal cysts are infrequent congenital malformations characterized by cystic dilatations of the biliary tree that was first described by Vater and Ezler in 1723 [1]. These dilatations can be extra-hepatic, intrahepatic or both. The incidence of choledochal cysts in Asian population is 1 in 1000 live births [2], 1:100,000 to 1:150,000 in the United States and 1 in 2 million in the United Kingdom. In Africa, there is insufficient data documented about choledochal cysts. More than 60 % of choledochal cysts present in the 1st year of life and 20 % in adulthood [3].

Initial classification by Alonso-Lej et al. in 1959 described 3 types of choledochal cysts, type I-III [4]. In 1977, Todani and others modified the original Alonso-Lej classification to include type IV and V with Type I further sub-classified into 3 types [5]. Choledochal cysts in children and adults behave differently with type I being common in children and type IV in adults [6]. A choledocal cyst that measures more than 10 cm is referred to as a giant cyst [5]. These are very rare and only a few have been reported in literature. Being rare makes it difficult to make diagnosis when encountered for the first time but also their size presents difficulties to the surgery which should be a complete excision. We report a case of a 17 year old female who was diagnosed and successfully managed for a giant choledochal cyst type IA at a Regional Referral Hospital in Uganda and has been reported in line with the SCARE 2020 criteria [7].

## 2 Case presentation

The 17 year old female presented with a 4 months history of constant, dull, non-radiating abdominal pain and a right upper quadrant mass progressively increasing in size. This was associated with yellowish discoloration of eyes, urine and clay colored stools plus one episode of vomiting but no history of diarrhea, fever or weight loss. There was no significant past medical or surgical history. One month prior to admission, she reported to have visited a peripheral lower level health facility that referred her to the regional referral but delayed to come due to financial constraints.

**Fig. 1:**
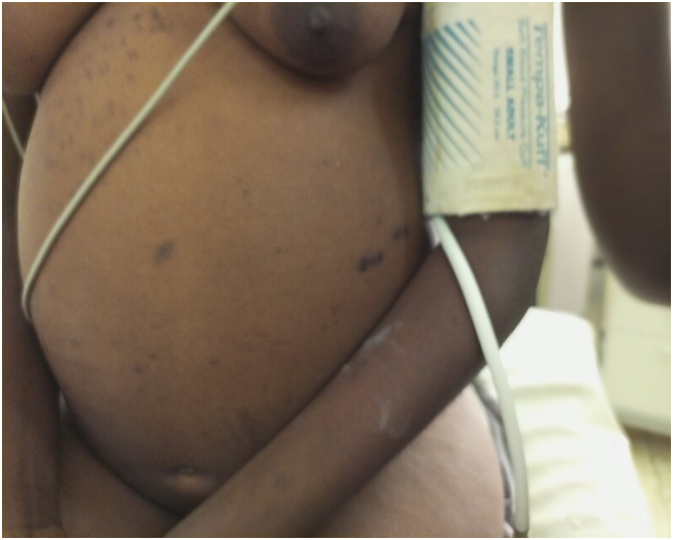
Pre-operative view of the distended abdomen.

On physical examination, she was in a fair general condition, well nourished, with obvious deep jaundice. Abdominal exam revealed a distended abdomen (Fig. 1) with a large palpable mass extending from the epigastrium to the hypogastrium. The mass was non tender, soft, non-mobile, couldn't go above it or below it and measured 30 cm × 20 cm. There were no visible collateral vessels, no inguinal masses or palpable lymph nodes.

The deranged laboratory measurements included total bilirubin (6.41 mg/dL), direct bilirubin (4.8 mg/dL), Alanine Transaminase (144 u/L), Aspartate Transaminase (208 u/L), Alkaline phosphatase (1827 u/L), Pro-thrombin time (18 s) and International Normalized Ratio (INR) (1.72). Complete blood count, renal function tests, serum electrolytes, hepatitis surface antigen and HIV test were unremarkable. CT scan revealed a huge cystic mass in the right upper quadrant extending inferiorly to the right lumbar region, surrounded by a thin enhancing wall on intravenous contrast. It was continuous with common bile duct and was compressing the gall bladder, head of pancreas and the right kidney. There was no focal or diffuse masses seen on the liver and there was no regional lymphadenopathy.

**Fig. 2:**
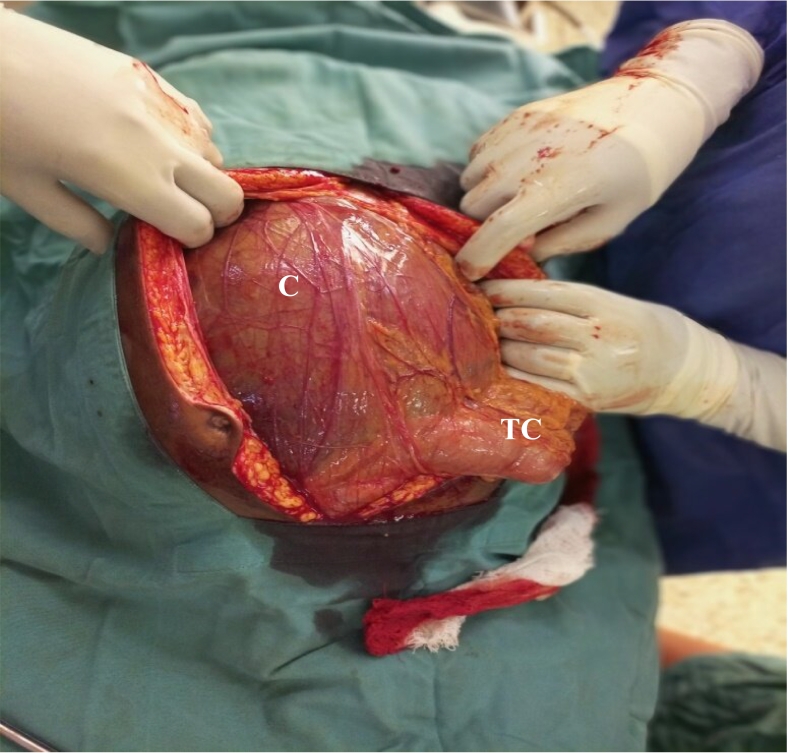
Intraoperative image of the Cyst (C) with adherent transverse colon (TC).

**Fig. 3:**
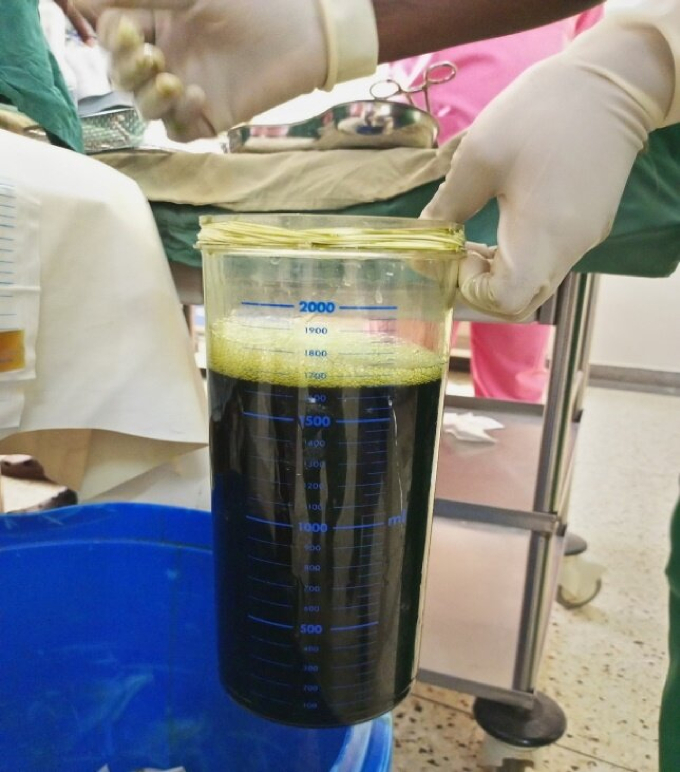
Bile content from the biliary cyst.

Laparotomy through an extended midline incision was done by a general surgeon. The laparotomy revealed a grossly enlarged cystic mass extending from the liver up to the hypo-gastric region with the duodenum, the gall bladder, greater omentum and transverse colon adherent onto the cystic mass (Fig. 2). Adhesions were carefully divided, separating the duodenum and transverse colon from the cystic mass while achieving hemostasis. Dissection continued medially and laterally to release the attachments of the cyst from other intra-abdominal wall structures. The mass was 30 cm by 25 cm by 20 cm containing over 2 L of greenish, bilious fluid (Fig. 3). The Cystic duct was identified and anterograde cholecystectomy was performed. A loop of jejunum 45 cm from the ligament of treitz was brought near the stump of common hepatic duct through a defect created on the left side of the middle colic vessels in the transverse mesocolon. Hepaticojejunostomy and jejunojejunostomy was done. A drain was left in proximity to the site of biliary reconstruction and was removed after it stopped draining. The specimen was sent for histo-pathological examination and was reported to contain chronic inflammatory infiltrate with no evidence of malignancy. Patient was discharged on the 7th post-operative day. At two months post operation, the patient had recovered completely when reviewed at the regional referral hospital and the liver function tests had normalized.

## 3 Discussion

Choledochal cysts are infrequent congenital malformations characterized by cystic dilatations of the biliary tree [1]. Giant choledochal cyst refer to the huge cysts with a diameter of greater than 10 cm [5]. For our case, intra-operatively, the cyst measured 30 cm by 25 cm by 20 cm which could be the largest reported in literature to the best of our knowledge. No strong unifying etiological theory exits for choledochal cysts but some series published by Miyayo and yamataka in 1977, documented an anomalous junctions in 90–100 % of patients with choledochal cysts [8]. This abnormal communication allows the mixing of bile and pancreatic juices activating pancreatic pro-enzymes that later damage and weaken the bile duct wall resulting in formation of choledochal cysts [9].

The primary diagnostic modality is by abdominal ultrasound scan followed by CT scan and the MRI. The associated anomalies are biliary atresia, gallbladder atresia, hepatic fibrosis and those of pancreatico-biliary ductal system. ERCP and MRCP have a conclusive role in confirming ultrasound scan diagnosis. These are also done to evaluate anatomy and identify complications related to the cyst [5]. In our case, the only imaging modalities available were abdominal ultrasound scan and the CT-scan. The abdominal ultrasound sound scan was used to screen the patient and identify the choledocal cyst. The CT-Scan was used to confirm our diagnosis and classify our cyst under Tonadi classification diagnosing it as type IA choledochal cyst.

The choledochal cyst is usually associated with complications that range from cholecystitis, cholangitis, pancreatitis, stone formation and malignancy. Prevalence of biliary malignancy is found to be around 30 % of the cases, this increase with age and is commonly higher in those with type I and type IV choledochal cysts [10]. Though our patient had a type 1 cyst and was 17 years old, it is fortunate that there was no malignancy.

The treatment of choledochal cyst is an initial control of complications followed by definitive surgery and reconstruction. Presently the most popular and accepted surgical intervention is by total excision of the choledochal cyst and restoration of the biliary enteric drainage either by doing hepaticoduodenostomy or Roux-en-Y hepatico-jejunostomy [11]. The later approach was used in our patient to reconstruct a connection between the common hepatic duct and a loop of jejunum 40 cm from the ligament of treitz. A laparoscopic approach is also currently being explored since it offers a less invasive surgical approach for the management of choledochal cysts in children [12], however these modalities are not yet available in most of the resource limited settings like the regional referral hospital where this patient was managed. Mores so, laparoscopic approach is more appropriate for cysts less than 7 cm [13] and ideal for narrow spaces such as esophageal hiatus and pelvic hiatus [14] and therefore may have not been appropriate in our patient. African countries are in dire need of adopting evolving technologies in order to improve surgical care [15].

## 4 Conclusion

Giant choledochal cyst is a very rare disease that may present both a diagnostic and surgical challenge. To the best of our knowledge, this is the largest giant Choledochal cyst reported in literature. Even in resource limited settings, sonography and a CT scan may be all that is required to make a diagnosis. Complete surgical excision reduces the chances of pancreatitis and biliary malignant transformation. During surgical excision, the surgeon should take extra caution to carefully dissect the adhesions off the giant cyst for a successful complete excision.

## Consent for publication

Written consent was obtained from the patient's guardian for publication of this case report and accompanying images and is available for the review by the Editor-in-Chief of this journal on request. Assent by the patient was also obtained.

## Ethical approval

This case report is exempted from ethical approval.

## Funding

This case report did not receive any specific grant from funding agencies in public, commercial, or not for profit sectors.

## Author contribution

**Oscar Atwine** managed the patient and wrote the first draft. **Charles Newton Odongo**, **Edson Tayebwa** and **Carlos Cabrera Dreque** were involved in patient management, review and editing of the paper, **Racheal Ainomugisha** and **Joshua Muhumuza** critically revised and edited the manuscript. All authors approved the final manuscript submitted.

## Research registration number

Not applicable.

## Guarantor

Oscar Atwine.

## Declaration of competing interest

No conflict of interest.
